# The Optimal Range of Serum Uric Acid for Cardiometabolic Diseases: A 5-Year Japanese Cohort Study †

**DOI:** 10.3390/jcm9040942

**Published:** 2020-03-30

**Authors:** Masanari Kuwabara, Ichiro Hisatome, Koichiro Niwa, Petter Bjornstad, Carlos A. Roncal-Jimenez, Ana Andres-Hernando, Mehmet Kanbay, Richard J. Johnson, Miguel A. Lanaspa

**Affiliations:** 1 Intensive Care Unit and Department of Cardiology, Toranomon Hospital, Tokyo 105-8470, Japan; 2 Cardiovascular Center, St. Luke’s International Hospital, Tokyo 104-8560, Japan; kniwa@aol.com; 3 Division of Regenerative Medicine and Therapeutics, Tottori University Graduate School of Medical Sciences, Tottori 683-8503, Japan; hisatome@med.tottori-u.ac.jp; 4 Division of Renal Diseases and Hypertension, School of Medicine, University of Colorado Denver, Aurora, CO 80045, USA; Petter.bjornstad@childrenscolorado.org (P.B.); Carlos.Roncal@ucdenver.edu (C.A.R.-J.); Ana.AndresHernando@ucdenver.edu (A.A.-H.); Richard.Johnson@ucdenver.edu (R.J.J.); Miguel.LanaspaGarcia@ucdenver.edu (M.A.L.); 5 Children’s Hospital Colorado and Barbara Davis Center for Childhood Diabetes, Aurora, CO 80045, USA; 6 Division of Nephrology, Department of Medicine, Koc University School of Medicine, Istanbul 34450, Turkey; mkanbay@ku.edu.tr

**Keywords:** uric acid, risk factor, epidemiology, cardiometabolic diseases, hypertension

## Abstract

The optimal range of serum uric acid (urate) associated with the lowest risk for developing cardiometabolic diseases is unknown in a generally healthy population. This 5-year cohort study is designed to identify the optimal range of serum urate. The data were collected from 13,070 Japanese between ages 30 and 85 at the baseline (2004) from the Center for Preventive Medicine, St. Luke’s International Hospital, Tokyo. We evaluated the number of subjects (and prevalence) of those free of the following conditions: hypertension, diabetes, dyslipidemia, and chronic kidney disease (CKD) over 5 years for each 1 mg/dL of serum urate stratified by sex. Furthermore, the odds ratios (ORs) for remaining free of these conditions were calculated with multiple adjustments. Except for truly hypouricemic subjects, having lower serum urate was an independent factor for predicting the absence of hypertension, dyslipidemia, and CKD, but not diabetes. The OR of each 1 mg/dL serum urate decrease as a protective factor for hypertension, dyslipidemia, and CKD was 1.153 (95% confidence interval, 1.068–1.245), 1.164 (1.077–1.258), and 1.226 (1.152–1.306) in men; 1.306 (1.169–1.459), 1.121 (1.022–1.230), and 1.424 (1.311–1.547) in women, respectively. Moreover, comparing serum urate of 3–5 mg/dL in men and 2–4 mg/dL in women, hypouricemia could be a higher risk for developing hypertension (OR: 4.532; 0.943–21.78) and CKD (OR: 4.052; 1.181–13.90) in women, but not in men. The optimal serum urate range associated with the lowest development of cardiometabolic diseases was less than 5 mg/dL for men and 2–4 mg/dL for women, respectively.

## 1. Introduction

Both epidemiologically and in animal models, hyperuricemia is strongly associated with the development and progression of cardiovascular disease [1,2]. In this regard, a recent meta-analysis report estimated a 12% increase in coronary heart disease per 1 mg/dL elevation in serum uric acid (urate) levels. However, it is important to note that other studies could not support serum urate as a truly independent risk factor for cardiovascular diseases [3,4,5]. Furthermore, some studies have shown a J-shaped relationship between serum urate and cardiovascular disease supporting the idea that both low and high urate are associated with greater risk of developing cardiovascular events. As an example, according to the PIUMA study, serum urate levels lower than 4.5 mg/dL in men and 3.2 mg/dL in women with essential hypertension but also higher than 5.2 mg/dL in men and 3.9 mg/dL in women are associated with increased rates of cardiovascular disease and cardiovascular disease-related deaths. The J-curve phenomenon relating serum urate to cardiovascular events was also reported in the Syst-Eur trial [6]. The variability in the findings from the cross-sectional studies and the evidence supporting a J-shaped cause-effect between urate and cardiovascular disease suggests the need to identify the proper level range for which urate could be relatively safe, or in contrast, could exert a substantial deleterious role in the pathogenesis of cardiovascular disease. In this regard, little information is known on the optimal serum urate range associated with the lowest risk of disease in a generally healthy population.

Therefore, we aimed to evaluate whether a J-shaped curve exists for serum urate with cardiometabolic diseases (hypertension, diabetes, dyslipidemia, and chronic kidney disease (CKD)) and to identify the optimal range of serum urate that is associated with the lowest risk for developing these conditions by a longitudinal design.

## 2. Materials and Methods

### 2.1. Study Design and Study Subjects

**Figure 1:**
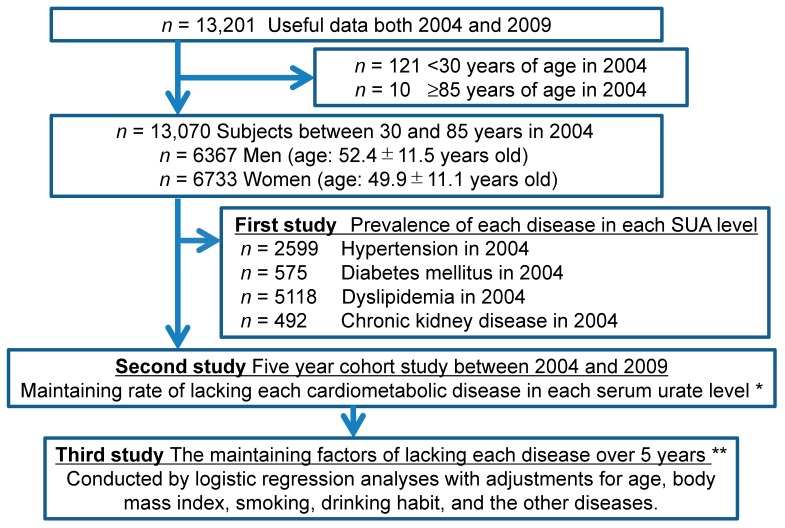
Flow diagram of study enrollment. All the analyses were stratified by sex. * Each cardiometabolic disease means hypertension, diabetes, dyslipidemia and chronic kidney disease. ** The number of subjects depends on the excluded subjects having the corresponding disorders at baseline.

This study included a single-center, large-scale, cross-sectional study and a 5-year longitudinal cohort study in Japan. We reviewed and used the database of health records from the Center for Preventive Medicine, St. Luke’s International Hospital, Tokyo, Japan between 2004 and 2009. The study subjects came to the center to have annual regular health check-up by themselves, and also provided a general history for comorbidities. Every subject and/or their companies paid for the examinations and each subject had identical physical and laboratory examinations including blood pressure. In general, the patients with any symptoms go to their hospital or clinic with Japanese government insurance, and therefore this study population was defined as a ‘generally healthy population’. Some of the findings from this database have already been published [7,8,9,10,11,12,13,14,15]. There were 30,227 subjects (15,263 men) who underwent an annual health check-up at the center in 2004. In this study, we enrolled 13,201 subjects who underwent health checks both in 2004 and in 2009. The background demographics between all the subjects in 2004 and this cohort study subjects were similar as shown in our previous manuscript [8]. The prevalence of hyperuricemia between the two groups showed no significant differences, including in men and in women, respectively [8]. For this study, we included subjects between the ages 30 and 85 in 2004 whose data were available at both 2004 and 2009. Subjects younger than 30 years of age were excluded due to their very modest risk for hypertension and cardiovascular diseases, while subjects aged 85 years old and above were excluded due to the substantial risk of death during a five-year follow-up. Out of the 13,201 subjects, only 121 subjects were less than 30 years old and 10 subjects were 85 years old and above in 2004, and 13,070 subjects were enrolled (mean age: 51.1 ± 11.3 years). Of these, 6367 were men (52.4 ± 11.5 years) and 6733 women (49.9 ± 11.1 years). For our first analysis (a cross-sectional study), we checked the prevalence of cardiometabolic diseases, like hypertension, diabetes, dyslipidemia, and CKD in each 1 mg/dL of serum urate range in each sex at baseline (2004). Then, we excluded the subjects with each cardiometabolic condition at baseline, and we checked whether they remained free of these conditions over the following five years, as it related to each serum urate quartile separated by sex (a 5-year cohort study). We also calculated the odds ratios (ORs) of each 1 mg/dL increase of serum urate for each cardiometabolic diseases after multiple adjustments for well-known factors associated with cardiovascular diseases including age, body mass index (BMI), smoking and drinking habits, serum urate, and the presence of other cardiometabolic diseases as detailed in Figure 1. When we assessed the protective factors for the development of hypertension, diabetes, dyslipidemia, and CKD, we excluded 2599 subjects with hypertensions, 575 subjects with diabetes, 5118 subjects with dyslipidemia, and 492 subjects with CKD at the baseline in each analysis, respectively. Moreover, we compared the cumulative incidence of any of these cardiometabolic diseases between hypouricemic subjects (serum urate of less than 3 mg/dL in men and less than 2 mg/dL in women) and normouricemic subjects (serum urate of 3–5 mg/dL in men and 2–4 mg/dL in women) to detect whether hypouricemia is a risk for developing cardiometabolic diseases compared with normouricemia. We also calculated the ORs of hypouricemia for each cardiometabolic diseases compared with normouricemia after multiple adjustments.

### 2.2. Definition of Hypertension, Diabetes, Dyslipidemia, CKD, and Hypouricemia

Hypertension is defined as a condition when subjects are on current antihypertensive medication and/or systolic blood pressure of more than or equal to 140 mmHg and/or diastolic blood pressure of more than or equal to 90 mmHg [16,17]. Blood pressure readings were obtained using an automatic brachial sphygmomanometer (OMRON Corporation, Kyoto, Japan), which was upper arm blood pressure measuring and had passed validation. Two blood pressure examinations were taken after the participants were seated and rested quietly for more than five minutes with their feet on the ground and their back supported. The mean systolic and diastolic blood pressure of each of the subjects were calculated from the recorded measurements. Diabetes is defined as current diabetes mellitus on medication use and/or HbA1c (National Glycohemoglobin Standardization Program) more than or equal to 6.5%, according to International Expert Committee. Dyslipidemia is defined as current medication use for dyslipidemia and/or low-density lipoprotein cholesterol more than or equal to 140 mg/dL, high-density lipoprotein cholesterol less than 40 mg/dL, and/or triglyceride more than or equal to 150 mg/dL, according to Japan Atherosclerosis Society guidelines [18]. CKD is defined as estimated glomerular filtration rate (eGFR) is less than 60 mL/min/1.73m^2^. We calculated eGFR using the Japanese GFR equation: eGFR (mL/min/1.73m^2^) = 194 × serum creatinine ^−1.094^ × age ^−0.287^ (×0.739 if woman) [19]. Hypouricemia is defined as serum urate level lower than 3.0 mg/dL in men and 2.0 mg/dL in women in this study [20]

### 2.3. Statistical Analysis

All the statistical analyses were performed using the SPSS Statistics software (IBM SPSS Statistics version 22 for Windows; IBM, New York, NY, USA). The statistically significant level was set at probability *p* < 0.05 (two-tailed). Data are expressed as mean ± standard deviation or as percent frequency unless otherwise specified. Comparisons between two groups were performed with student *t*-tests for normally distributed variables, and χ^2^ analyses for categorical data. The maintaining factors for lacking hypertension, diabetes, dyslipidemia, and CKD in the period of over five years were evaluated both by crude models and by multivariable logistic regression models with adjustments of the age, BMI, smoking and drinking habits, serum urate, and the other cardiometabolic diseases. We also calculated odds ratios (ORs) in each group. When we analyzed logistic regression analyses in the longitudinal study, we excluded hypouricemic subjects because there was not a linear association between serum urate levels and the maintaining rate of lacking prevalence of these cardiometabolic diseases only in hypouricemic subjects. Moreover, we compared cumulative incidence of each cardiometabolic disease between hypouricemia and normouricemia to clarify whether *J.* curve phenomenon exists or not. In this analysis, we used propensity score matching to combine the other factors (age, BMI, smoking and drinking habits, and cardiometabolic feathers; hypertension, diabetes, dyslipidemia, and CKD) into one parameter because the number of hypouricemic subjects were small (45 hypouricemic subjects).

### 2.4. Ethical Considerations

We adhered to the principles of the Declaration of Helsinki. All data were collected and compiled in a protected computer database. Individual data were anonymous without identifiable personal information. Informed consent was obtained from all subjects by a comprehensive agreement method provided by St. Luke’s International Hospital. St. Luke’s International Hospital Ethics Committee approved the protocol for this study (approval number: 16-R025).

## 3. Results

### 3.1. Demographics of this Study’s Subjects

Table 1 shows the demographics of this study for men and women. In general, women were significantly older, and had lower BMI, lower blood pressure, less smoking and drinking habits, lower prevalence of hypertension, diabetes, dyslipidemia, and CKD, and lower serum urate compared to men.

### 3.2. Prevalence of Cardiometabolic Disease in Each Serum Urate Level (A Cross-Sectional Study)

**Figure 2:**
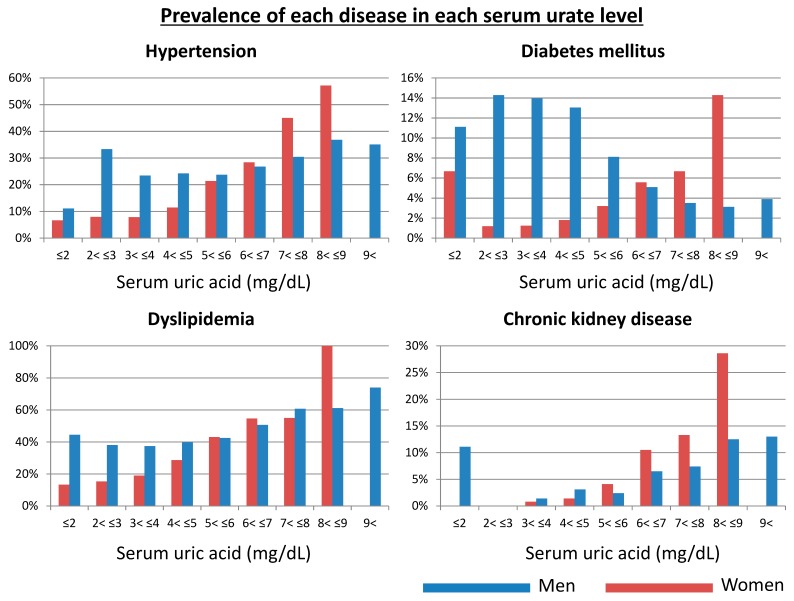
Prevalence of hypertension, diabetes, dyslipidemia, and chronic kidney disease in each serum urate at baseline (2004). Blue bars showed men and red bars showed women.

Figure 2 shows the prevalence of hypertension, diabetes, dyslipidemia, and CKD for each 1mg/dL of serum urate range at baseline (2004). As shown in the figure, serum urate lower than 4 mg/dL were associated with the lowest prevalence of hypertension, dyslipidemia, and CKD in women while the range of serum urate between 2 and 4 mg/dL corresponded with the lowest prevalence of diabetes. In men, the range of serum urate associated with the lowest prevalence of these conditions was more variable. Interestingly, the prevalence of diabetes in men decreased with increasing serum urate, which may be due to the effect of glycosuria to cause uricosuria and decrease serum urate levels. As a result, serum urate levels in men ranging from 2 to 6 mg/dL corresponded with the lowest prevalence of dyslipidemia and CKD while levels ranging from 3 to 6 mg/dL were associated with the lowest prevalence of hypertension, respectively. However, it is important to note that this cross-sectional analysis at baseline did not account for medication for each disease, raising the possibility of a potential medication bias. Therefore, we conducted a 5-year cohort study to evaluate the odds for remaining free of these disease conditions over time.

### 3.3. Rate of Being Free of Various Cardiometabolic Conditions According to Serum Urate Levels over Five Years (A Longitudinal Study)

**Figure 3:**
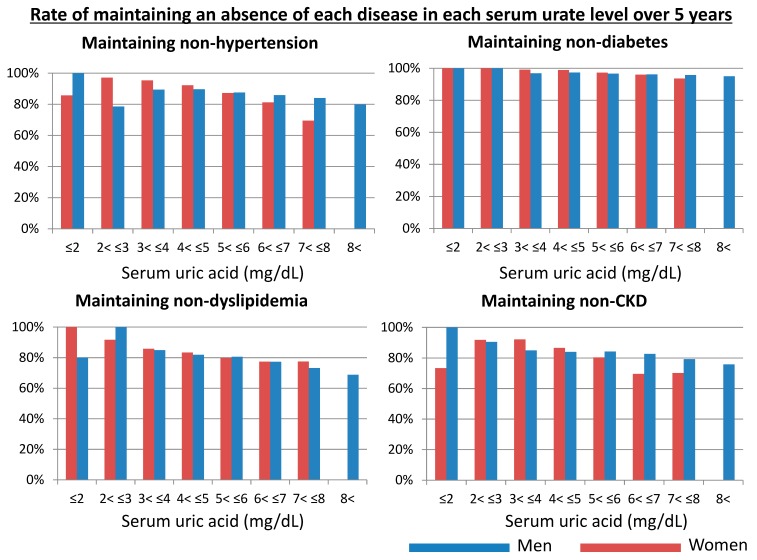
Maintaining rate of lacking hypertension, diabetes, dyslipidemia, and chronic kidney disease in each serum urate over five years. CKD, chronic kidney disease. Blue bars showed men and red bars showed women.

The number of subjects with the new development of hypertension, diabetes, dyslipidemia, and CKD over 5 years was 1108/10,471 (10.6%), 318/12,495 (2.5%), 1454/7952 (18.3%), and 1961/12,578 (15.6%), respectively. Figure 3 shows the relative risk for being free of cardiometabolic disease (hypertension, diabetes, dyslipidemia, and CKD) for each serum urate group over a five-year period. There is a linear association between serum urate levels and the rate of being free of cardiometabolic disease except for subjects with hyporuricemia. and we excluded hypouricemic subjects. The multivariable analyses showed that serum urate levels were protective for developing hypertension, diabetes, dyslipidemia, and CKD irrespective of sex, except for hypouricemic subjects. Even when accounting for a J-curve phenomenon with hypouricemic subjects, hyperuricemic subjects had lower maintaining rates with respect to lacking hypertension, diabetes, dyslipidemia, and CKD than hypouricemic subjects.

**Figure 4:**
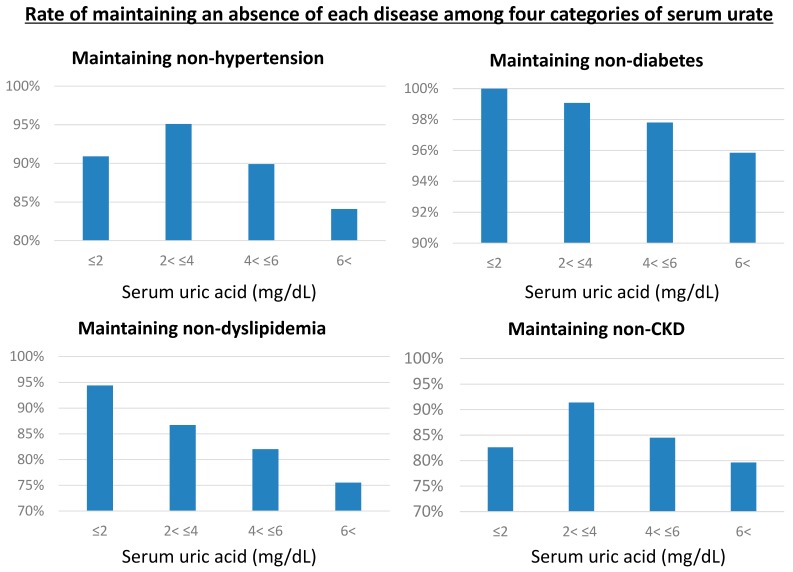
Maintaining rate of lacking hypertension, diabetes, dyslipidemia, and chronic kidney disease among four serum urate categories over five years. CKD, chronic kidney disease.

We conducted additional analyses using four categories of serum urate levels; 2 or less (hypouricemia), from 2 to 4 mg/dL, from 4 to 6 mg/dL, and more than 6 mg/dL (hyperuricemia). We compared the relative risk for being free of cardiometabolic disease (hypertension, diabetes, dyslipidemia, and CKD) among these four serum urate categories over a five-year period. Figure 4 shows that the group with serum urate from 2 to 4 mg/dL exhibited maintaining rates of lacking hypertension or CKD compared to the other urate categories. The group with serum urate of 2 mg/dL or less had the highest maintaining rate with respect to lacking diabetes or dyslipidemia.

### 3.4. Optimal Serum Urate Range Associated with the Lowest Risk of Cardiometabolic Diseases

To determine the optimal range of serum urate to prevent the development of cardiometabolic disease, we conducted a multivariable logistic regression analysis and calculated ORs for maintaining conditions without hypertension, diabetes, dyslipidemia, and CKD after excluding 45 hypouricemic subjects (30 men and 15 women) since we intended to exclude the effects of *J.* curve phenomenon.

To evaluate factors that predict continued normotension, we analyzed 10,471 subjects after excluding 2599 subjects with hypertensions at baseline. After multivariable adjustments for age, BMI, smoking and drinking habits, diabetes, dyslipidemia, and CKD, lower serum urate was an independent factor that protects against the development of hypertension both in men (OR per 1 mg/dL decrease: 1.153; 95% CI, 1.068–1.245) and women (OR: 1.306; 95% CI, 1.169–1.459) (Table 2, Hypertension).

When we assessed the factors that protected against the development of diabetes, we analyzed 12,495 subjects after excluding 575 subjects with diabetes at the baseline. After multiple adjustments age, BMI, smoking and drinking habits, hypertension, dyslipidemia, and CKD, lower serum urate showed a tendency as a protective factor for the development of diabetes in women (OR per 1 mg/dL decrease: 1.206; 95% CI, 0.969–1.500), but it did not reach the significant (*p* = 0.093). In contrast, lower serum urate was not an independent protective factor for the development of diabetes in men (*p* = 0.24) (Table 2, Diabetes).

When we assessed the protective factor for the development of dyslipidemia, we analyzed 7952 subjects after excluding 5118 subjects with dyslipidemia at baseline. After multiple adjustments age, BMI, smoking and drinking habits, hypertension, diabetes, and CKD, lower serum urate was an independent protective factor for the development of dyslipidemia both in men (OR per 1 mg/dL decrease: 1.164; 95% CI, 1.077–1.258) and women (OR per 1 mg/dL decrease: 1.121; 95% CI, 1.022–1.230) (Table 2, Dyslipidemia).

When we assessed the factors that protected against the development of CKD, we analyzed 12,578 subjects after excluding 492 subjects with CKD at the baseline. After multiple adjustments age, BMI, smoking and drinking habits, hypertension, diabetes, and dyslipidemia, lower serum urate was an independent protective factor for the development of CKD both in men (OR per 1 mg/dL decrease: 1.226; 95% CI, 1.152–1.306) and women (OR per 1 mg/dL decrease: 1.424; 95% CI, 1.311–1.547) (Table 2, Chronic kidney disease).

We also compared the ORs for hypertension, diabetes, dyslipidemia, and CKD among four categories of serum urate levels. We referenced the group with serum urate from 2 to 4 mg/dL as shown in Table 3. Belonging to the group with serum urate from 2 to 4 mg/dL conferred protection from developing hypertension, dyslipidemia, and CKD when compared with the group with serum urate more than 4 mg/dL, but not diabetes (Table 3).

### 3.5. Hypouricemia as a Risk of Cardiometabolic Diseases Compared with Normouricemia

We compared the cumulative incidence of cardiometabolic diseases between hypouricemic subjects (30 men and 15 women) and normouricemic subjects (3–5 mg/dL for 958 men and 2–4 mg/dL for 2192 women). The number of hypouricemic subjects were small, and we could not analyze ORs of diabetes both in men and women and dyslipidemia in women. After multiple adjustments age, BMI, smoking and drinking habits, diabetes, dyslipidemia, and CKD, hypouricemia tends to be higher risk for the development of CKD in women (OR: 4.532; 95% CI, 0.943–21.78), but not reach significance (*p* = 0.059) (Table 4, Hypertension). After multiple adjustments that included age, BMI, smoking and drinking habits, hypertension, diabetes, and CKD, hypouricemia continued to show a higher risk for the development of CKD in women (OR: 4.052; 95% CI, 1.181–13.90), but not in men. (Table 4, Chronic kidney disease). The cumulative incidence of hypertension, dyslipidemia, and CKD in men was not significantly different between hypouricemic and normouricemic groups.

## 4. Discussion

The primary goal of our study was to identify the range of serum urate associated with the lowest risk for developing cardiometabolic diseases in a healthy Japanese population. Except for truly hypouricemic subjects (defined as ≤3 mg/dL in men and ≤2 mg/dL in women), our study indicates that lower serum urate level is an independent protective factor for the development of cardiometabolic disease. We show that in heathy subjects, for each 1 mg/dL decrease of serum urate in men, there was an 18% increment in the protection from developing hypertension, a 16% increment against dyslipidemia, and a 23% increment against CKD. Compared to men, lower serum urate in women conferred greater odds for preventing the appearance of cardiometabolic diseases. Specifically, for each 1 mg/dL decrease of serum urate in women, there was a 31% increment in the protection from developing hypertension, a 12% increment against dyslipidemia, and a 42% increment against CKD.

We also compared the cumulative incidence of cardiometabolic diseases over 5 years between hypouricemic subjects and normouricemic subjects (3–5 mg/dL for men and 2–4 mg/dL for women). The number of hypouricemic subjects was small (30 men and 15 women), and it might be difficult to apply the results to every population because of less power to analyze. However, our results suggest that hypouricemia could be a risk for development of hypertension and CKD in women, but not in men. Accounting for these results, we could see the *J.* curve phenomenon only in women, and the optimal serum urate range associated with the less development of cardiometabolic diseases could be less than 5 mg/dL for men and 2–4 mg/dL for women in a generally healthy population.

Other studies have also showed an inverse correlation between serum urate and the incidence of cardiovascular diseases in subjects with serum urate levels lower than 4.5 mg/dL in men and 3.2 mg/dL in women [6,21,22]. This phenomenon is observed primarily in those subjects with low serum urate levels. Of note, the study subjects in these previous reports often were hypertensive, diabetic or receiving medication against these conditions [6,21,22]. In our study, we can see the similar J-curve phenomenon in hypertension and CKD in women, but the serum urate levels required for this J-shape phenomenon were much lower compared to those reported in previous studies [6,21,22]

Our study also showed that hypouricemic subjects demonstrated greater risk for developing hypertension and CKD than normouricemic subjects in women. We postulate that the higher cumulative incidence of cardiometabolic diseases in hypouricemic women could well relate to the relatively frequent genetic loss of the urate transporter (URAT) in the Japanese population. Potential mechanisms for why this increases the risk for these conditions might relate to the marked uricosuria that may increase the risk for kidney disease, or potentially the possibility that a low serum urate may reduce antioxidant activity in the patients [23,24]. Importantly, there are no studies to determine whether lowering serum urate levels to very low levels with xanthine oxidase inhibitors increases cardiovascular risk compared to untreated controls.

Our study points out the necessity of addressing the risk of hypouricemia in addition to hyperuricemia in the pathogenesis of cardiovascular disease. Our published data demonstrated that hypouricemia is associated with endothelium dysfunction [23]. Consistently, a large-scale cross-sectional study showed that hypouricemic men had higher rates of kidney disease compared to non-hypouricemic subjects. However, the rates of other diseases including diabetes and urinary stones were not significantly different between hypouricemic and non-hypouricemic subjects [20]. In this regard, our longitudinal study showed that hypouricemia did not carry the lowest risk for developing cardiometabolic diseases. Since excess serum urate not only has an adverse effect, but also acts preferably as a reducing substance, this dual nature needs to be considered clinically.

This study showed a positive association between serum urate and cardiometabolic diseases, but most Mendelian randomization studies or meta-analyses suggested that elevated serum urate was only associated with gout [25,26,27,28,29]. However, Mendelian studies are often limited by not considering other influencing conditions, such as life habits including food, alcohol, and fructose intake. Most hyperuricemia is mainly acquired by life habits except for some genetic diseases [30], and it is therefore difficult to apply the results from Mendelian studies to most acquired hyperuricemic subjects. The gap of results between clinical studies and genetic studies suggest that acquired hyperuricemia may cause more cardiometabolic diseases than genetic hyperuricemia.

Our study has several limitations. First, this study is a retrospective single center study, which may have introduced selection bias. However, single center studies had some advantages of the similarity of the methodology. Second, we could not check the additional and withdrawal medication or gouty attacks over the periods. Some hyperuricemic subjects with gouty attacks might have medication especially non-steroidal anti-inflammatory drugs (NSAIDs), which might cause CKD or hypertension. However, our definition of each disease included medication use. Moreover, we did not exclude the subjects on medication for hyperuricemia or gout intentionally, because the serum urate levels on medication could be useful to evaluate the effects of serum urate on cardiometabolic diseases. However, there is a possibility of the influence of urate-lowering medications on the development (or prevention) of other cardiometabolic diseases, which thus may bias the present results. We additionally conducted the sensitivity analyses that excluded 373 (2.9%) subjects with urate-lowering medications (Supplementary Table S1). The results showed the same results, thus supporting our main results more robustly. However, this study was not able to show whether urate-lowering medications could prevent cardiometabolic diseases or not because this study was an observational study. We had to adjust the patient backgrounds between the medication group and the control group to show the efficacy of urate-lowering medications for hyperuricemia to prevent cardiometabolic diseases. Third, this longitudinal study lacks time-to-event data, which precluded survival analysis. Fourth, we measured serum urate only once, and blood pressure only at the center. Serum urate can fluctuate for natural or iatrogenic causes. Moreover, some hypertensive subjects might have white-coat hypertension and some non-hypertensive subjects might have masked hypertension. Measuring serum urate many times and ambulatory blood pressure monitoring are the best to evaluate serum urate and blood pressure precisely, but it is difficult in practice in the setting of an annual medical examination. Fifth, the number of hypouricemic subjects was small, and it might be less power to analyze the significant difference. Therefore, it is difficult to discuss the J-curve phenomenon precisely. Finally, causality cannot be inferred, because this is an observational study. Interventional studies are needed to further clarify whether the treatments for hyperuricemia are useful for preventing the development of cardiometabolic diseases.

## 5. Conclusions

Even in the normal range, having higher serum urate could be a risk for hypertension, dyslipidemia, and CKD. The optimal serum urate range, which conferred the lowest risk for developing cardiometabolic diseases, could be less than 5 mg/dL for men and 2–4 mg/dL for women in a generally healthy population. These findings suggest that routine screening of serum urate is useful as a predictor for cardiometabolic diseases in primary care settings.

## Figures and Tables

**Table 1:** Demographics of study subjects at baseline (2004).

	Women	Men	*p*
Number of Subjects	6733	6337	
Age	49.9 ± 11.1	42.4 ± 11.5	<0.001
Body mass index (kg/m^2^)	21.3 ± 3.0	23.8 ± 2.9	<0.001
Systolic blood pressure (mmHg)	114.5 ± 17.5	124.2 ± 17.2	<0.001
Diastolic blood pressure (mmHg)	70.8 ± 10.9	77.9 ± 10.9	<0.001
Pulse rate (bpm)	75.2 ± 10.8	71.6 ± 10.5	<0.001
Smoking	16.3%	63.0%	<0.001
Drinking habits	26.0%	61.5%	<0.001
Hypertension	13.4%	26.8%	<0.001
Diabetes mellitus	2.1%	6.8%	<0.001
Dyslipidemia	29.6%	49.3%	<0.001
Hypouricemia	0.22%	0.47%	0.016
Chronic kidney disease	2.3%	5.3%	<0.001
eGFR (mL/min/1.73m^2^)	88.2 ± 15.7	82.6 ± 15.5	<0.001
Serum uric acid (mg/dL)	4.49 ± 0.95	6.24 ± 1.23	<0.001

bpm, beats per minute; *p*, probability. Data are presented as mean ± standard deviation.

**Table 2:** Lower serum urate as a protective factor for the development of hypertension, diabetes, dyslipidemia and chronic kidney disease over 5 years.

Maintaining without Hypertension			Crude			Adjusted *		
**Women**			OR	95% CI	*p*	OR	95% CI	*p*
Serum uric acid	per 1 mg/dL decreased		1.755	1.587–1.941	<0.001	1.306	1.169–1.459	<0.001
**Men**								
Serum uric acid	per 1 mg/dL decreased		1.180	1.099–1.266	<0.001	1.153	1.068–1.245	<0.001
**Maintaining without diabetes mellitus**			**Crude**			**Adjusted †**		
**Women**			OR	95% CI	*p*	OR	95% CI	*p*
Serum uric acid	per 1 mg/dL decreased		1.822	1.507–2.202	<0.001	1.206	0.969–1.500	0.093
**Men**								
Serum uric acid	per 1 mg/dL decreased		1.160	1.037–1.298	0.010	1.074	0.953–1.210	0.24
**Maintaining without dyslipidemia**			**Crude**			**Adjusted ‡**		
**Women**			OR	95% CI	*p*	OR	95% CI	*p*
Serum uric acid	per 1 mg/dL decreased		1.311	1.202–1.429	<0.001	1.121	1.022–1.230	0.015
**Men**								
Serum uric acid	per 1 mg/dL decreased		1.205	1.120–1.296	<0.001	1.164	1.077–1.258	<0.001
**Maintaining without chronic kidney disease**			**Crude**			**Adjusted** ¶		
**Women**			OR	95% CI	*p*	OR	95% CI	*p*
Serum uric acid	per 1 mg/dL decreased		1.655	1.545–1.795	<0.001	1.424	1.311–1.547	<0.001
**Men**								
Serum uric acid	per 1 mg/dL decreased		1.144	1.082–1.210	<0.001	1.226	1.152–1.306	<0.001

OR, odds ratio; CI, confidence interval; *p*, probability. * Data adjusted for age, body mass index, smoking and drinking habits, diabetes mellitus, dyslipidemia, chronic kidney disease, and serum uric acid. † Data adjusted for age, body mass index, smoking and drinking habits, hypertension, dyslipidemia, chronic kidney disease, and serum uric acid. ‡ Data adjusted for age, body mass index, smoking and drinking habits, hypertension, diabetes mellitus, chronic kidney disease, and serum uric acid. ¶ Data adjusted for age, body mass index, smoking and drinking habits, hypertension, diabetes mellitus, dyslipidemia, and serum uric acid.

**Table 3:** Lower serum urate as a protective factor for the development of hypertension, diabetes, dyslipidemia and chronic kidney.

Maintaining without hypertension	Crude			Adjusted *		
Serum uric acid	OR	95% CI	*P*	OR	95% CI	*p*
2 mg/dL to 4 mg/dL	Reference			Reference		
2 mg/dL and less	1.922	0.444–8.328	0.38	1.705	0.385–7.564	0.48
4 mg/dL to 6 mg/dL	2.170	1.756–2.682	<0.001	1.543	1.237–1.926	<0.001
more than 6 mg/dL	3.630	2.920–4.512	<0.001	2.031	1.570–2.628	<0.001
**Maintaining without diabetes mellitus**	**Crude**			**Adjusted** †		
Serum uric acid	OR	95% CI	*P*	OR	95% CI	*p*
2 mg/dL to 4 mg/dL	Reference			Reference		
2 mg/dL and less	–	–	–	–	–	–
4 mg/dL to 6 mg/dL	2.404	1.530–3.779	<0.001	1.405	0.881–2.238	0.153
more than 6 mg/dL	4.634	2.957–7.262	<0.001	1.571	0.947–2.606	0.080
**Maintaining without dyslipidemia**	**Crude**			**Adjusted** ‡		
Serum uric acid	OR	95% CI	*P*	OR	95% CI	*p*
2 mg/dL to 4 mg/dL	Reference			Reference		
2 mg/dL and less	0.384	0.051–2.896	0.35	0.346	0.046–2.622	0.30
4 mg/dL to 6 mg/dL	1.437	1.233–1.674	<0.001	1.259	1.073–1.478	0.005
more than 6 mg/dL	2.116	1.784–2.508	<0.001	1.568	1.267–1.940	<0.001
**Maintaining without chronic kidney disease**	**Crude**			**Adjusted** ¶		
Serum uric acid	OR	95% CI	*p*	OR	95% CI	*p*
2 mg/dL to 4 mg/dL	Reference			Reference		
2 mg/dL and less	2.236	0.754–6.634	0.15	2.368	0.752–7.459	0.14
4 mg/dL to 6 mg/dL	1.949	1.665–2.281	<0.001	1.579	1.337–1.864	<0.001
more than 6 mg/dL	2.716	2.307–3.199	<0.001	2.345	1.927–2.854	<0.001

OR, odds ratio; CI, confidence interval; *p*, probability. * Data adjusted for age, body mass index, smoking and drinking habits, diabetes mellitus, dyslipidemia, chronic kidney disease, and serum uric acid. † Data adjusted for age, body mass index, smoking and drinking habits, hypertension, dyslipidemia, chronic kidney disease, and serum uric acid. ‡ Data adjusted for age, body mass index, smoking and drinking habits, hypertension, diabetes mellitus, chronic kidney disease, and serum uric acid. ¶ Data adjusted for age, body mass index, smoking and drinking habits, hypertension, diabetes mellitus, dyslipidemia, and serum uric acid.

**Table 4:** Hypouricemia (serum urate less than 3 mg/dL in men and less than 2 mg/dL in women) as a risk factor for the development of hypertension, diabetes, dyslipidemia and chronic kidney disease over 5 years compared with normouricemia (serum urate of 3–5 mg/dL in men and 2–4 mg/dL in women).

Hypertension		Crude			Adjusted *		
**Women**	**Reference**	OR	95% CI	*p*	OR	95% CI	*p*
Hypouricemia (*n* = 14)	Normouricemia (*n* = 2020)	3.659	0.807–16.599	0.093	4.532	0.943–21.78	0.059
**Men**							
Hypouricemia (*n* = 22)	Normouricemia (*n* = 728)	1.355	0.392–4.684	0.545	1.141	0.319–4.075	0.84
**Diabetes**		**Crude**			**Adjusted**		
**Women**	**Reference**	OR	95% CI	*p*	OR	95% CI	*p*
Hypouricemia (*n* = 14)	normouricemia (*n* = 2165)	–			–		
**Men**							
Hypouricemia (*n* = 26)	normouricemia (*n* = 831)	–			–		
**Dyslipidemia**		**Crude**			**Adjusted †**		
**Women**	**Reference**	OR	95% CI	*p*	OR	95% CI	*p*
Hypouricemia (*n* = 13)	normouricemia (*n* = 1789)	–			–		
**Men**							
Hypouricemia (*n* = 18)	normouricemia (*n* = 582)	0.28	0.037–2.129	0.219	0.238	0.031–1.847	0.17
**Chronic kidney disease**		**Crude**			**Adjusted ‡**		
**Women**	**Reference**	OR	95% CI	*p*	OR	95% CI	*p*
Hypouricemia (*n* = 15)	normouricemia (*n* = 1795)	4.212	1.327–13.37	0.015	4.052	1.181–13.90	0.026
**Men**							
Hypouricemia (*n* = 29)	normouricemia (*n* = 932)	0.396	0.093–1.681	0.209	0.303	0.068–1.351	0.117

OR, odds ratio; CI, confidence interval; *p*, probability; N/A, not available for analysis. * Data adjusted for age, body mass index, smoking and drinking habits, diabetes mellitus, dyslipidemia, chronic kidney disease, and serum uric acid. † Data adjusted for age, body mass index, smoking and drinking habits, hypertension, diabetes mellitus, chronic kidney disease, and serum uric acid. ‡ Data adjusted for age, body mass index, smoking and drinking habits, hypertension, diabetes mellitus, dyslipidemia, and serum uric acid.

## Supplementary Materials

The following are available online at https://www.mdpi.com/2077-0383/9/4/942/s1, Table S1. The sensitivity analyses that excluded 373 (2.9%) subjects with urate-lowering medications: Lower serum.
