# Supplementary material for: The Optimal Range of Serum Uric Acid for Cardiometabolic Diseases: A 5-Year Japanese Cohort Study
Source: J Clin Med. 2020 Mar 30;9(4):942. doi: 10.3390/jcm9040942 (PMC7231289; doi:10.3390/jcm9040942)
Supplement: Supplementary file 1 [file jcm-09-00942-s001.pdf]

Supplementary Table S1. The sensitivity analyses that excluded 373 (2.9%) subjects with urate-lowering medications: Lower serum urate as a protective factor for the development of hypertension, diabetes, dyslipidemia and chronic kidney disease over 5 years.

**Maintaining without hypertension**		**Crude**			**Adjusted***		
**Women**		OR	95% CI	P	OR	95% CI	P
Serum uric acid	per 1 mg/dL decreased	1.760	1.591-1.947	<0.001	1.309	1.171-1.462	<0.001
**Men**							
Serum uric acid	per 1 mg/dL decreased	1.173	1.090-1.262	<0.001	1.151	1.063-1.246	<0.001

**Maintaining without diabetes mellitus**		**Crude**			**Adjusted†**		
**Women**		OR	95% CI	P	OR	95% CI	P
Serum uric acid	per 1 mg/dL decreased	1.835	1.516-2.220	<0.001	1.206	0.969-1.502	0.093
**Men**							
Serum uric acid	per 1 mg/dL decreased	1.172	1.039-1.321	0.010	1.08	0.950-1.227	0.24

**Maintaining without dyslipidemia**		**Crude**			**Adjusted‡**		
**Women**		OR	95% CI	P	OR	95% CI	P
Serum uric acid	per 1 mg/dL decreased	1.317	1.207-1.436	<0.001	1.127	1.027-1.237	0.012
**Men**							
Serum uric acid	per 1 mg/dL decreased	1.190	1.104-1.282	<0.001	1.146	1.058-1.242	<0.001

Maintaining without chronic kidney disease		Crude			Adjusted¶		
**Women**		OR	95% CI	P	OR	95% CI	P
Serum uric acid	per 1 mg/dL decreased	1.660	1.540-1.791	<0.001	1.422	1.309-1.545	<0.001
**Men**							
Serum uric acid	per 1 mg/dL decreased	1.157	1.092-1.227	<0.001	1.245	1.166-1.329	<0.001

OR, odds ratio; CI, confidence interval; p, probability

*Data adjusted for age, body mass index, smoking and drinking habits, diabetes mellitus, dyslipidemia, chronic kidney disease, and serum uric acid.

†Data adjusted for age, body mass index, smoking and drinking habits, hypertension, dyslipidemia, chronic kidney disease, and serum uric acid.

‡Data adjusted for age, body mass index, smoking and drinking habits, hypertension, diabetes mellitus, chronic kidney disease, and serum uric acid.

¶Data adjusted for age, body mass index, smoking and drinking habits, hypertension, diabetes mellitus, dyslipidemia, and serum uric acid.
